# Comparison of earthquake-induced shallow landslide susceptibility assessment based on two-category LR and KDE-MLR

**DOI:** 10.1038/s41598-023-28096-z

**Published:** 2023-01-16

**Authors:** Xinyue Fan, Bin Liu, Jie Luo, Ke Pan, Suyue Han, Zhongli Zhou

**Affiliations:** 1 Geomathematics Key Laboratory of Sichuan Province, Chengdu, 610059 China; 2 College of Mathematics and Physics, Chengdu University of Technology, Chengdu, 610059 China; 3 College of Management Science, Chengdu University of Technology, Chengdu, 610059 China

**Keywords:** Natural hazards, Mathematics and computing

## Abstract

Geological hazards caused by strong earthquakes have caused continuous social and economic losses and destruction of the ecological environment in the hazard area, and are mostly manifested in the areas with frequent occurrence of geological hazards or the clustering of geological hazards. Considering the long-term nature of earthquakes and geological disasters in this region, this paper takes ten earthquake-stricken areas in Wenchuan earthquake zone as examples to collect shallow landslide data in 2010, combined with the spatial location of landslides and other factors. Kernel density estimation (KDE) method is used to analyze the spatial characteristics of shallow landslide. Taking the space of shallow landslide as the characteristic variable and fully considering the regulating factors of earthquake-induced landslide: terrain complexity, distance to river, distance to fault, distance to road, lithology, normalized vegetation difference index (NDVI) and ground peak acceleration (PGA) as independent variables, based on KDE and polynomial logistic regression (MLR), A quantitative model of shallow landslide in the earthquake area is constructed. The results show that: (1) PGA has the greatest impact on landslide in the study area. (2) Compared with the two-category logistic regression (two-category LR) model, the susceptibility map of landslide prediction results based on the KDE-MLR landslide susceptibility prediction model is more consistent with the actual situation. (3) The prediction accuracy of the model validation set is 70.7%, indicating that the landslide susceptibility prediction model based on KDE-MLR can effectively highlight the spatial characteristics of shallow landslides in 10 extreme disaster areas. The research results can provide decision-making basis for shallow landslide warning and post-disaster reconstruction in earthquake-stricken areas.

## Introduction

Earthquakes play a relatively prominent role in various natural hazards and are extremely destructive. There are many earthquakes every year around the world, and China's seismic fault zone is greatly affected by earthquake hazards. According to incomplete statistics, the cumulative death toll in China resulting from earthquakes that occurred between 1993 and 2016 accounted for 50% of global earthquake-related deaths. Moreover, more than two-thirds of China’s provinces were affected by earthquakes, resulting in millions of casualties and hundreds of millions of victims^1,2^. After a strong earthquake, the geological structure of the affected area becomes unstable and susceptible to geological hazards such as landslides, debris flows, dammed lakes, and avalanches that occur frequently in extremely earthquake-stricken areas^3^. Among these hazards, landslides are one of the most frequent and destructive earthquake-induced geological hazards in the world^4–6^. According to a large amount of data, losses caused by landslides have far exceeded losses from earthquakes themselves. This had a huge impact on the sustainable development of society and the economy^7^. The Wenchuan earthquake (M_W_-7.9) that occurred on the Longmenshan fault zone in 2008 was the most destructive earthquake in the past 100 years. The energy released was approximately three times that of the Tangshan earthquake. In addition, a post-earthquake emergency investigation of geological hazards showed that there was a total of 8060 hidden geological hazard points across 39 earthquake-stricken counties in Sichuan Province, fully demonstrating that the temporal and spatial effects of geological hazards after the Wenchuan earthquake were significant^8^.

Previous research on the evaluation of landslides, has mainly been conducted on three aspects: landslide sensitivity^9–12^, landslide risk^13–15^ and landslide susceptibility^16^. Among then, landslide susceptibility can be defined as the spatial probability of landslide occurrence based on a set of geological and environmental conditions. A landslide susceptibility map reflects the spatial distribution of the landslide probability in an area^16^. This analysis can be traced back to the Japanese scholar Saito, who, in the 1960s, used the results of creep tests to predict the locations of shallow landslides. After completing a series of related theoretical systems, many scholars have conducted exploratory research on other quantitative spatial prediction models of regional geological hazards, including empirical models (such as fuzzy logic and generalized additive models)^17,18^, statistical analysis models (such as the certainty coefficient method, weights of evidence and entropy models)^19–21^ and pattern recognition models (such as artificial neural networks, support vector machines (SVMs) and adaptive, neuro-fuzzy inference systems (ANFISs))^22–24^. Two-category LR is a generalized linear regression analysis model widely used in exploring landslide susceptibility due to the simple calculations and physical clear meaning characteristic. However, this model is relatively simple and cannot handle complex issues in actual situations, some scholars have proposed MLR. The MLR is useful in situations with several dependent variables^25^. Previous findings indicate that the two-category LR is widely used in shallow landslide susceptibility evaluations^26,27^. The dependent variables of most two-category LR in susceptibility analysis of shallow landslides are binary logic occurrence variables (denoted as 1) and nonoccurrence variables (represented as 0). Notably, the intensity of regional shallow landslides (spatial effects) is not considered in this model resulting in significant forecast biases.

Tobler's first law of geography states that everything is related to other things but near things are more related than distant things; that is, there is a potential dependence between the observed data of certain variables in the same or different distribution areas^28^. Landslides are spatially manifested as regions where geological hazards or groups of hazards frequently occur. In the past, the spatial quantitative modelling of landslides primarily comprised use of statistics to explore the characteristics and development of hazards (whether a hazard occurred or not). Although the spatial distribution of geological hazards appears random, it exhibits inherent regularity, thus spatial autocorrelation is widely used in the study of geological hazards^29,30^. Zou et al.^29^ established spatial regression models using the relevant geological hazard data, evaluated the impact of human activities on geological hazards in the Shennongjia Mountain area, and proposed effective prevention and control strategies. Liu et al.^31^ explored the spatial–temporal distribution and conditioning factors of geological hazards. Gonzalez et al.^32^ investigated the spatial distribution characteristics of seismic hazards in cities and the impact of river vaults on them using the conventional spatial autocorrelation method (SPAC) and Calicatas SPAC method. Previous findings indicate that evaluating the spatial characteristics of landslides improves the accuracy of spatial prediction of landslides.

The purpose of this paper is to build a model to predict shallow landslide susceptibility. The disaster intensity of regional shallow landslides was quantitatively characterized by the model, and an objective understanding of landslide spatial characteristics was used to accurately predict the influence of the landslide disasters. By identifying the shallow landslide susceptibility area, research on shallow landslide spatial characteristics can effectively reduce casualties and property losses. In this paper, ten earthquake-stricken areas affected by the Wenchuan earthquake are studied. The evaluation factors affecting the surface sensibility of shallow landslides are examined from the aspects of topography, geological environment and inducing conditions. KDE is used to describe the multiclassification spatial characteristics of shallow landslides, and the degree of aggregation and dispersion of shallow landslides in space is fully analyzed. A landslide disaster sensitivity prediction model based on KDE-MLR is constructed using MLR. The remainder of the paper is organized as follows: in "Introduction" Section, summarize the research progress of spatial quantitative modeling of landslides. In "Overview of the study area" Section, introduce the 10 severely stricken areas of the Wenchuan earthquake, and the data sources. In "Method" Section, introduce the research methods used in this paper, including: KDE, LR algorithm (two-category LR, MLR), Model performance evaluation index. In "Results" Section, construct the landslide susceptibility prediction model based on KDE-MLR, analyze the influence degree of landslide conditioning factors, evaluate the model and discuss the validity of the model. In "Discussion" Section, conclusions are presented. In "Conclusions" Section, discussion is presented.

## Overview of the study area

**Figure 1:**
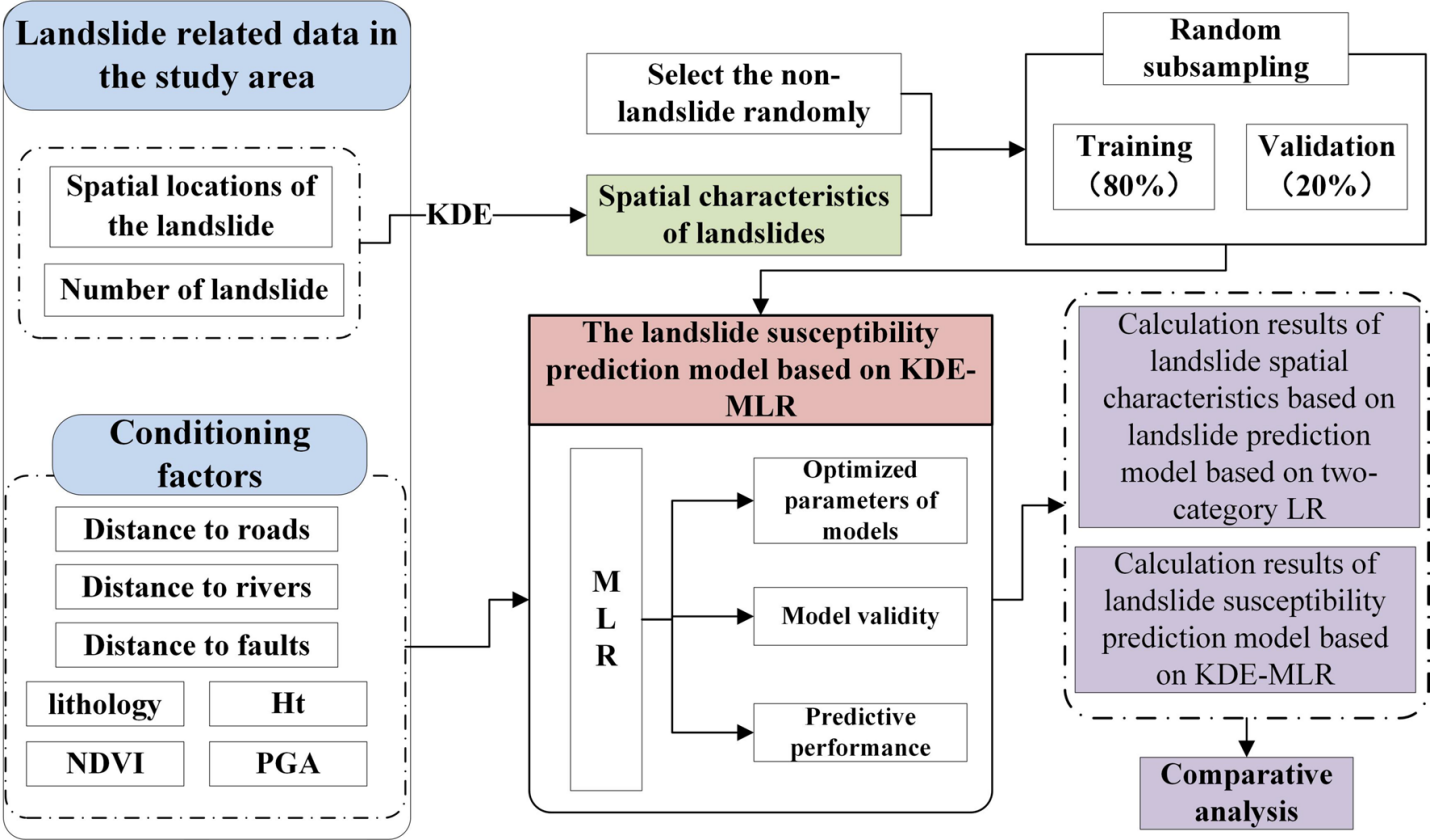
Methodological flowchart of the study.

This paper conducts a spatial prediction of landslides in the 10 extremely earthquake-stricken areas of the Wenchuan earthquake and improves the two types of dependent variables over those used in traditional landslide evaluations (1 for landslide occurrences and 0 for landslide non-occurrences). First, the spatial characteristics of landslides are analyzed by the KDE method. Second, by taking the spatial characteristics of landslides as the dependent variable, using the advantages of the MLR method, and expanding it into three categories, then construct a landslide susceptibility prediction model based on KDE-MLR. Finally, a spatial landslide susceptibility map is generated. The research framework, which is shown in Fig. 1 is divided into five steps:

- *Step 1* Collect landslide geological hazard data in the study area, including the spatial location of landslide points, the number of landslides, and related data on seven conditioning factors.
- *Step 2* Account for factors such as the spatial locations of the landslides, KDE is used to describe the spatial characteristics of the landslide in the study area.
- *Step 3* Use the MLR and take the spatial characteristics of landslides as dependent variables and the conditioning factors as independent variables, then construct a landslide susceptibility prediction model based on KDE-MLR.
- *Step 4* Randomly select 80% of the spatial characteristics of landslides in the study area as training samples and 20% as verification samples, then continuously adjust the model parameters and evaluate the effectiveness and performance of the model.
- *Step 5* Compare and analyze the susceptibility maps generated by the Landslide prediction model based on two-category LR and the landslide susceptibility prediction model based on KDE-MLR.

### Study area

**Figure 2:**
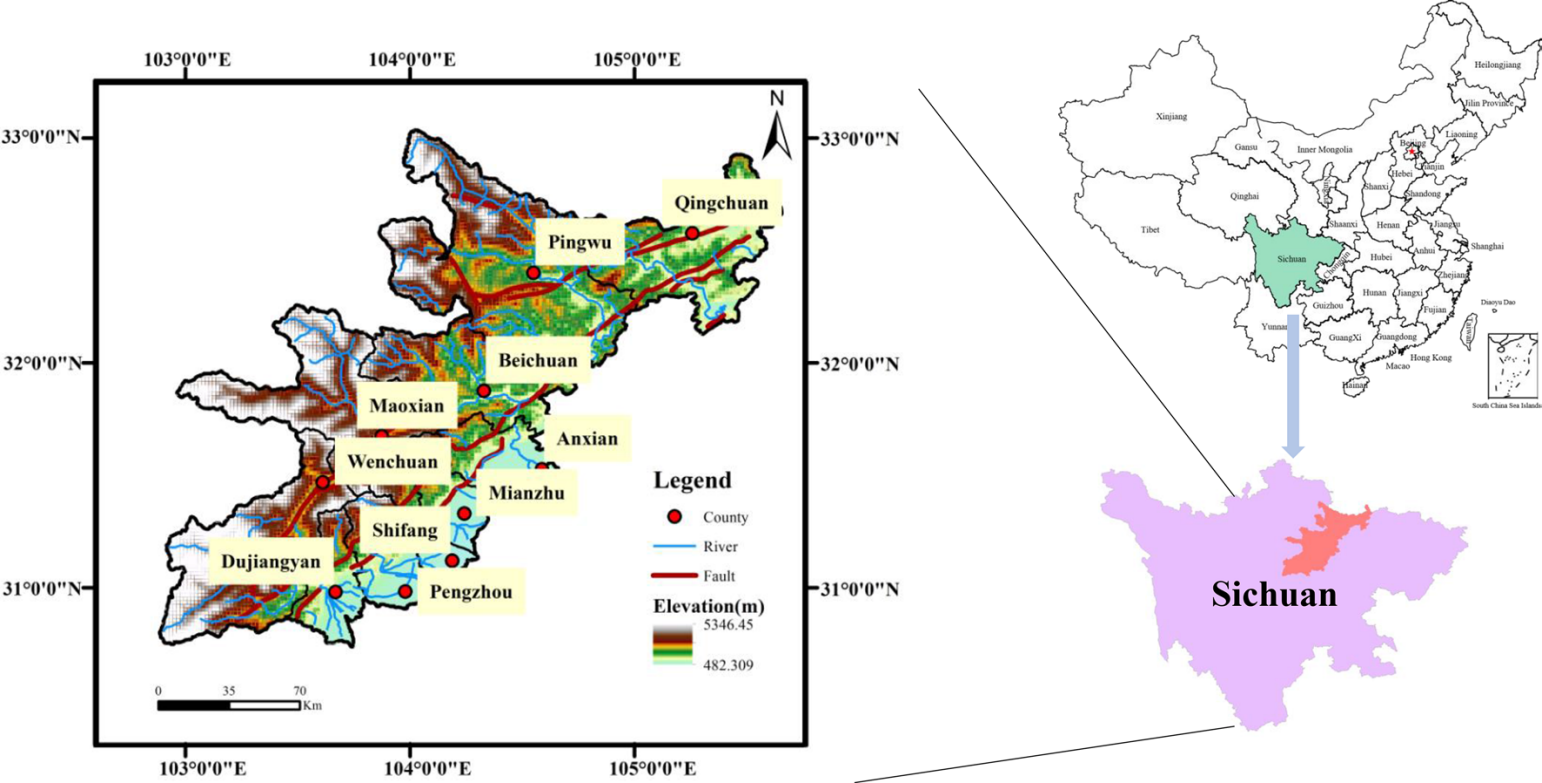
Map of the study area (Names of software: ArcGIS 10.5 and Office 2019).

In 2008, the 5.12 Wenchuan earthquake shook Sichuan, Gansu and Shaanxi provinces. This paper studies the 10 extremely earthquake-stricken areas of the Wenchuan earthquake that were announced by the Ministry of Land and Resources of China. These 10 extremely earthquake-stricken areas suffered the most damage during the earthquake and are all located in Sichuan Province: Wenchuan, Beichuan, Qingchuan, Maoxian, Anxian, Mianzhu, Shifang, Dujiangyan, Pingwu and Pengzhou (Fig. 2). The study area covers approximately 26,000 km^2^ and has a total population of over 3.5 million. The topography of the region is relatively complex, with elevations ranging between 490 and 5600 m, and the average annual precipitation is between 490 and 1400 mm^33^. In addition, the terrain in this area is complex, located in the Longmenshan fault zone (one of the fault zones with the largest topographic gradient in the world), and there have been many strong earthquakes. By investigating the occurrence of geological hazards (e.g., landslides) in the extremely earthquake-stricken areas of the Wenchuan earthquake, landslides can be found to have increased sharply 2–3 years after the earthquake^34^. Therefore, this paper takes 2010 as an example to analyze the spatial distribution characteristics of landslides in the 10 extremely earthquake-stricken areas and establishes a quantitative spatial landslide model to provide a reference for hazard monitoring and decision-making to formulate emergency plans.

### Data sources

#### Data preparation

**Figure 3:**
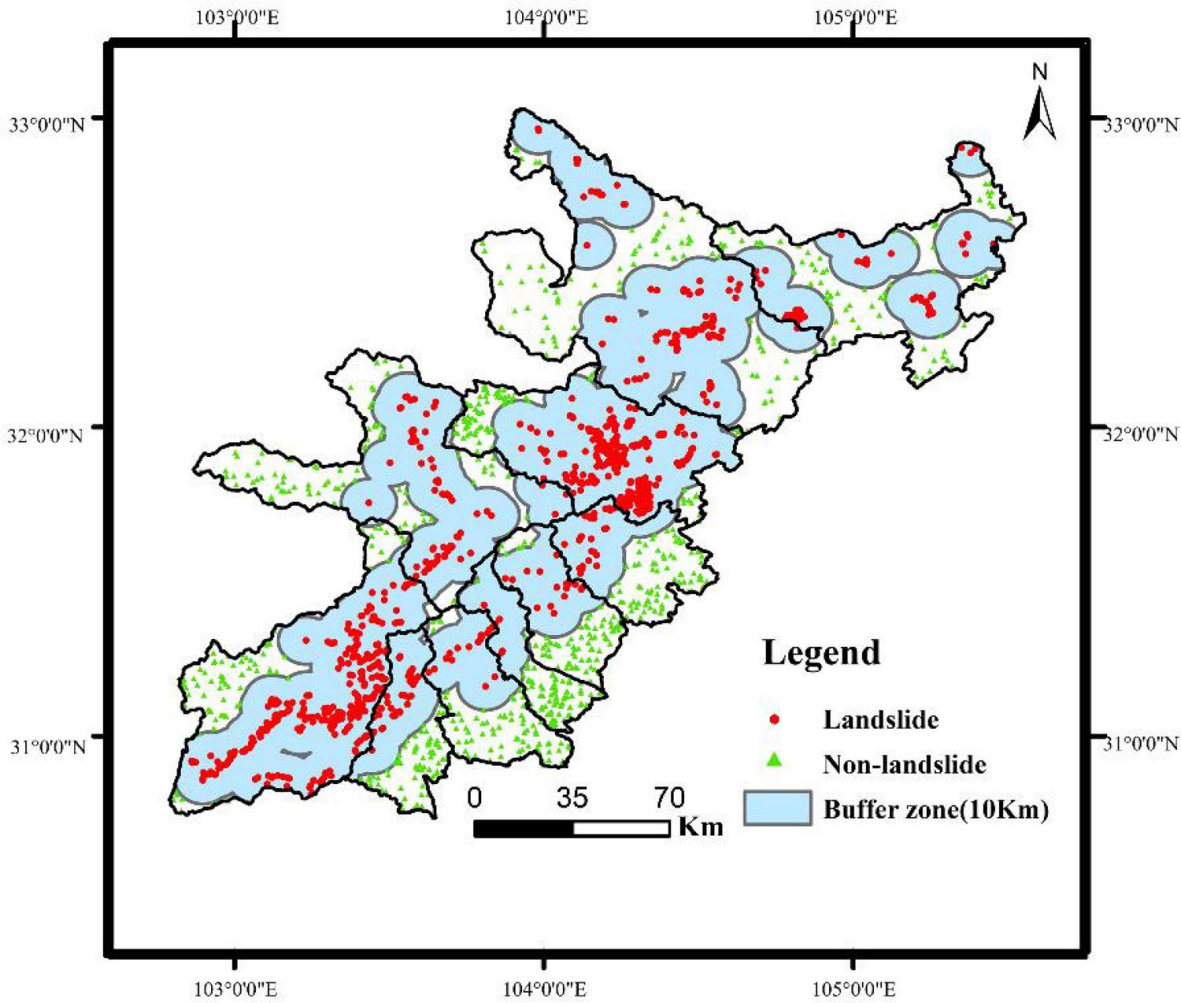
Spatial distribution of landslides (Name of software: ArcGIS 10.5).

In this study, the landslide that occurred in 2010 (2 years after the Wenchuan earthquake) was selected as the research object due to the long-term nature of the earthquake and geological hazards. The spatial location and number of shallow landslides in the study area of this paper were obtained by Landsat-8 30 m resolution remote sensing data interpretation, and grid processing was carried out with 60 m*60 m pixels in ArcGIS 10.5 software. According to the results of remote sensing analysis and statistics, there were 885 landslide points in the study area in 2010. However, in real life, the occurrence of shallow landslides often changes the whole area, which may make the model ignore the change in nonlandslide points in the calculation process. This reduces the accuracy of the landslide susceptibility evaluation map due to inconsistency with the actual situation. A total of 885 non-landslide points were randomly selected in a 1:1 ratio outside the buffer zone, 10 km away from the landslide point to reduce errors caused by this situation. The spatial distribution of the 885 non-landslide points is shown in Fig. 3.

#### Conditioning factors of landslides

**Figure 4:**
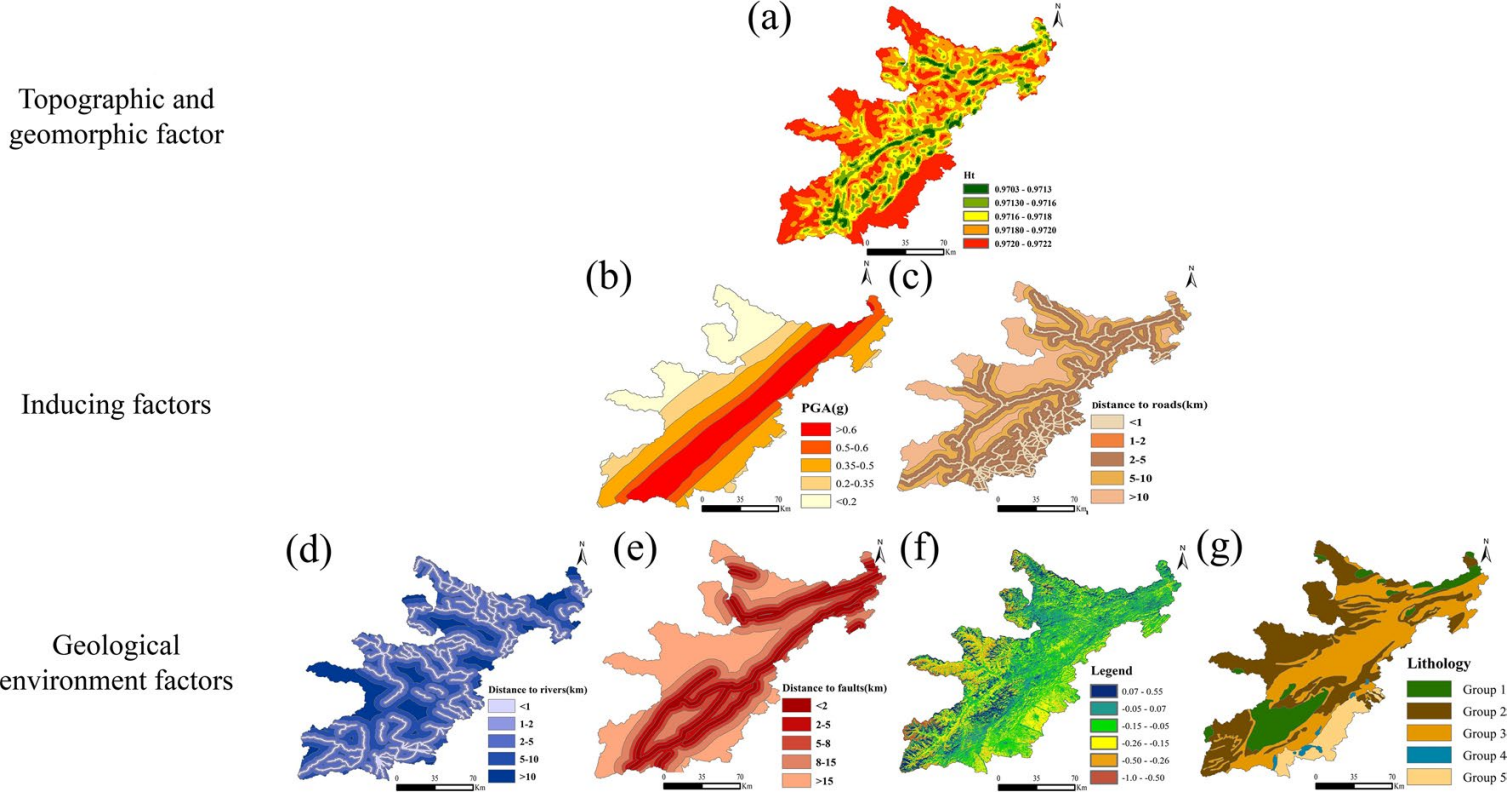
Conditioning factors of landslides: (**a**) Ht, (**b**) PGA, (**c**) distance to roads; (**d**) distance to rivers, (**e**) distance to faults, (**f**) NDVI, and (**g**) lithology (Names of software: ArcGIS 10.5 and Office 2019).

The selection of conditioning factors is one of the most important tasks in spatial predictive modelling^35^. Although there is currently no clear guideline for selecting conditioning factors when constructing prediction models, the selection of conditioning factors usually depends on the characteristics of the study area and the scale of the hazard^36,37^. For this paper, we selected seven landslide conditioning factors or independent variables based on the quality and availability of data: terrain information entropy (Ht) (Fig. 4a), PGA (Fig. 4b), distance to roads (Fig. 4c), distance to rivers (Fig. 4d), distance to faults (Fig. 4e), NDVI (Fig. 4f) and lithology (Fig. 4g). Among them, Ht is a topographic and geomorphic factor, distance to rivers, distance to faults, lithology, and NDVI are geological environment factors, and distance to roads and PGA are inducing factors.

All conditioning factors were generated using ArcGIS software. The topographic information entropy was calculated using 1:35 0000 DEM data^30^. The distance to the fault was derived from the 1:500,000 regional geological map^38^. The distance to the river was obtained from 1:3 million National Hydrogeological Atlas (https://www.osgeo.cn/map/m04dd). Statistical yearbook and historical data were obtained through remote sensing interpretation. Lithology data were retrieved from 1:500,000 regional geological map^38,39^. NDVI was obtained from Landsat-7 30-m resolution remote sensing interpretation. PGA data were retrieved from the United States Geological Survey (USGS) report. The 7 conditioning factor layers were gridded with 60 $\times$ 60 m pixels in ArcGIS 10.5 software, generating a total of 1,002,7131 grids, which are used as input data for generating the landslide susceptibility map, as shown in Fig. 4.

## Method

### Kernel density estimation (KDE)

In order to deeply reveal the spatial distribution characteristics of earthquake and landslide geological hazards, this paper selects the KDE to quantitatively calculate the spatial distribution characteristics of landslides, and analyzes the spatial continuous trend of landslides in the hardest hit area of the Wenchuan earthquake. KDE is mainly used to estimate the density of point or line features around each output raster cell^40^. Through the two-dimensional greyscale expression or three-dimensional surface expression of the earthquake-induced landslide core density calculation results, the distribution characteristics of the cluster or dispersion of the landslide point groups can be simply and intuitively obtained. The two-dimensional kernel density is an extension of the one-dimensional kernel density, and the density value at a certain point is estimated by calculating the kernel estimator:

1 $$f(s) = \frac{1}{nh2}\sum\limits_{i = 1}^{n} {K0} \left( {\frac{{\left| {dis} \right|}}{h}} \right)$$

In formula (1), $s$ is the spatial location of an estimated point, $n$ is the number of landslide points within the bandwidth, $d_{is}$ is the distance from the *i*_*th*_ landslide point to $s$, and $h$ is the bandwidth. The bandwidth formula is as follows:

2 $$h = 0.9 \times \min \left( {SD,\;\sqrt {\frac{1}{\ln (2)}} \times D_{m} } \right) \times N^{ - 0.2}$$

3 $$SD = \sqrt {\frac{{\sum\limits_{i = 1}^{M} {(x_{i} - \overline{X} )^{2} } }}{M} + \frac{{\sum\limits_{i = 1}^{M} {(y_{i} - \overline{Y} )^{2} } }}{M}}$$

In formulas (2) and (3), $D_{m}$ is the median distance from each landslide point to the average centre, $N$ is the sum of the landslide points, $SD$ is the standard distance, $M$ is the total number of landslide points, $x_{i}$ and $y_{i}$ are the coordinates of the landslide points $i$ and $\overline{X}$ and $\overline{Y}$ are the average center of the landslide points. $K_{0}$ is the kernel function, and its formula is shown:

4 $$K_{0} \left( {\frac{{\left| {d_{is} } \right|}}{h}} \right) = \left\{ \begin{gathered} \frac{3}{\pi }\left( {1 - \frac{{\left| {d_{is} } \right|^{2} }}{{h^{2} }}} \right)^{2} \quad 0 < d_{is} \le h \hfill \\ {0}\quad \quad \quad \quad \quad \quad \quad \quad d_{is} > h \hfill \\ \end{gathered} \right.$$

### LR algorithm

#### Two-category LR

The two-category LR method is a nonlinear, multivariate statistical method that is widely used to evaluate landslide susceptibility^41–43^. The two-category LR model can perform different types of independent variable analysis, including analyses of continuous variables and discrete variables. It comprehensively evaluates various conditioning factors based on actual landslide point samples and non-landslide point samples and can better solve the problem of factor interdependence^44,45^. The two-category LR method does not require the independent variables to conform to the normal distribution and it has no restriction on the distribution of the identified variables. It can be used to predict the probability of dependent variables with binomial characteristics. When the dependent variable in the two-category LR model is of two types, namely, occurrence of a landslide (denoted as 1) and nonoccurrence of a landslide (denoted as 0), the relationship between the probability of landslide occurrence and the conditioning factor is shown in the following formula^46^:

5 $$Z = a_0 + a_1x_1 + a_2x_2 + \cdots + a_ix_i$$

6 $$P = \frac{\exp (z)}{{1 + \exp (z)}}$$

In formulas (5) and (6), is the two-category LR prediction value representing the probability of landslide occurrence and having a value in the range [0, 1], $Z$ represents the sum of the linear weight values after the variables are superimposed, is the constant term of the logistic regression, $xi$ represents each conditioning factor and is the two-category LR coefficient of the *i*th conditioning factor.

#### MLR

In practical problems, the dependent variable may have multiple values. When there are more than two dependent variable values, MLR analyses need to be used. MLR analysis is a variant of two-category LR and its basic concept is essentially the same as that of two-category LR^47,48^. MLR analysis assumes that dependent variable categories are completely independent. It also takes one of the categories as a reference group and calculates a reference group regression coefficient^49^.

The MLR model can be considered a *J*-1 two-category LR model of J dependent variables and each independent dependent variable is compared with the reference group to calculate the advantage of the reference group^50^. Assuming that Y = *J* is the reference group, the LR model of each category is as follows:

7 $$\lambda j{ = }\log \left( {\frac{Pj}{{PJ}}} \right) = \alpha j + \beta 1jx1j + \cdots \beta ijxij$$

In formula (7), $j = {1}, \ldots J - {1}$, $\alpha j$ is the intercept, $xi$ represents each conditioning factor, and $\beta ij$ is the MLR coefficient of the No. *i*_*th*_ conditioning factor. The MLR model is expressed in the form of probability as follows:

8 $$Pj = \frac{\exp (\lambda j)}{{1 + \sum\limits_{i = 1}^{J - 1} {\exp (\lambda j)} }}$$

9 $$PJ = \frac{1}{{1 + \sum\limits_{i = 1}^{J - 1} {\exp (\lambda j)} }}$$

In formulas (8) and (9), $Pj$ represents the probability that the dependent variable is a certain category and $PJ$ represents the probability of the reference group. The values of the dependent variable Y in this study are 0, 1 and 2. Since most of the locations in the 10 extremely earthquake-stricken areas are non-landslide points, the dependent variable Y = 0 is set as the reference group.

### Model performance evaluation index

To evaluate the performance of both the two-category LR and KDE-MLR landslide susceptibility prediction models, this paper uses an error matrix to evaluate the accuracy of each model^51^, as shown in Table 1. The overall accuracy is principally used to determine the performance of the models and is obtained by comparing the predicted model category with the actual category.

**Table 1:** Error matrix used for the accuracy assessment.

Actual category	Forecast category		
	0	1	2
0	**N1**	N2	N3
1	N4	**N5**	N6
2	N7	N8	**N9**

0, 1, 2 are dependent variables respectively; N1 means the actual category is 0, the forecast category is 0, and so on.

In MLR analysis, training sets are used to train the model. When predicting the remaining test datasets, the probability of each category is compared by calculating the predicted dependent variable probabilities for different categories and the reference group. The highest category is the result of the final classification^50^. The effectiveness of the model is determined by its overall accuracy rate, which expresses the accuracy of the model. The accuracy rate is the ratio of the number of samples that predict the correct classification of the model to the total number of samples. The specific formula is as follows:

10 $$Overall\;accuracy = \frac{N1 + N5 + N9}{{\sum\limits_{i = 1}^{9} {Ni} }}$$

## Results

### Calculation results of the spatial characteristics of landslides

**Figure 5:**
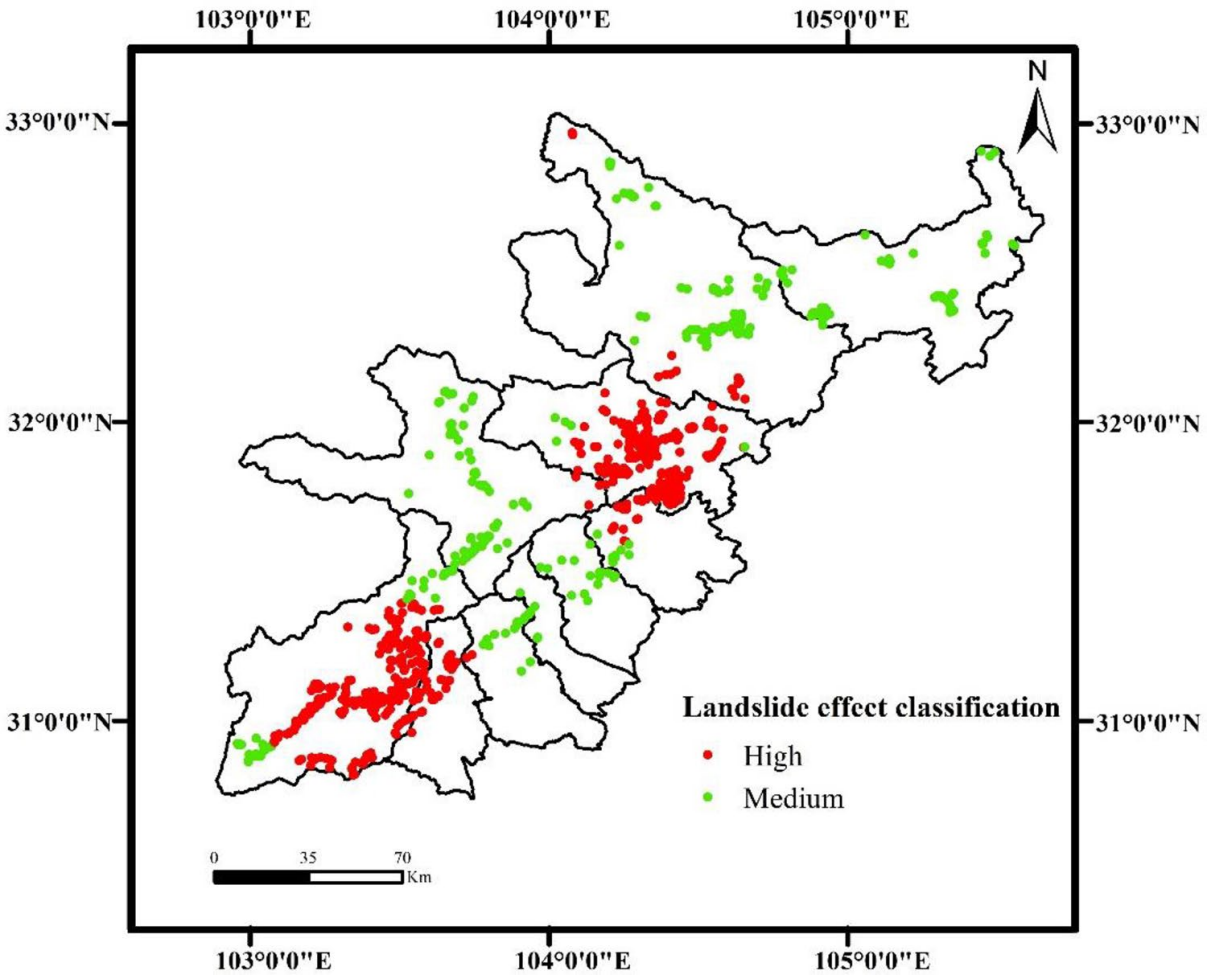
Calculation results of the spatial characteristics of landslides (Name of software: ArcGIS 10.5).

To further understand the spatial distribution characteristics of landslides, this paper comprehensively considers the spatial location and number of landslides. This paper also uses the KDE formula in Sect. Kernel density estimation (KDE) to calculate the spatial characteristics of landslides of the 10 extremely earthquake-stricken areas, as shown in Fig. 5.

Based on the value of spatial distribution characteristics that calculated by KDE, this paper uses the average value (3.3) as the demarcation point to divide the spatial characteristics of landslides into two levels (High, Medium). Thus, the spatial characteristics of landslides of all landslide points is divided into 2 levels, High and Medium. In Fig. 5, approximately 62% of the landslide points have high spatial aggregation of landslides. These points mainly lie in Wenchuan and Beichuan. The KDE value of 337 landslide disaster points in the entire study area is lower than 3.3, the landslide spatial characteristics is moderate, and the distribution in other counties (cities) is relatively uniform.

### Analysis of the influence degree of factors on landslides

#### Analysis of landslide conditioning factors based on LR

In the two-category LR landslide prediction model, this paper randomly selects 80% of the 885 landslide and non-landslide points (619 landslide points and 619 non-landslide points) as the training set and 20% (266 landslide points and 266 non-landslide points) as the validation set. Two-category LR is performed based on the extracted landslide conditioning factors and training samples, and the regression coefficient of each conditioning factor is obtained. As shown in Table 2, the greater the absolute value of the coefficient, the greater the impact it has on landslide susceptibility.

**Table 2:** Two-category LR coefficient results.

Serial number	1	2	3	4	5	6	7
Conditioning factor	Distance to roads	PGA	Distance to rivers	Distance to faults	NDVI	Ht	Lithology
Absolute value of the standardized regression coefficient	0.0575	0.418	0.3663	0.008	0.052	0.2611	0.381

Table 2 shows that the absolute value of the PGA regression coefficient is the highest (0.418), which indicates that this factor has the largest influence on earthquake-induced landslides. The lithology, distance to rivers and Ht also have relatively large impacts on landslides in the study area, with absolute values of their regression coefficients between 0.2 and 0.4. The regression coefficients of the distance to roads, NDVI and distance to faults are all less than 0.1, indicating that these three conditioning factors have relatively little correlation with landslides.

#### Analysis of landslide conditioning factors under KDE-MLR

In the landslide susceptibility prediction model based on KDE-MLR, the dependent variable (the spacial characteristics the of landslides) is a categorical variable and the independent variable (conditioning factor) is a categorical, or continuous, variable in the MLR analysis^52^. MLR uses different criteria in determination of the MLR coefficients of discrete variables and continuous variables. Therefore, continuous variables were classified in this study and treated as separate variables (Table 3).

**Table 3:** Classification of Influential Factors.

Serial number	Conditioning factor	Grading situation	Classification based on
1	Distance to roads	Level 1: ≤ 1 km; Level 2: 1 ~ 2 km; Level 3: 2 ~ 5 km; Level 4: 5 ~ 10 km; Level 5: ≥ 10 km	^53^
2	Distance to faults	Level 1: ≤ 2 km; Level 2: 2 ~ 5 km; Level 3: 5 ~ 8 km; Level 4: 8 ~ 15 km; Level 5: ≥ 15 km	^54^
3	Distance to rivers	Level 1: ≤ 1 km; Level 2: 1 ~ 2 km; Level 3: 2 ~ 5 km; Level 4: 5 ~ 10 km; Level 5: ≥ 10 km	^55^
4	PGA	Level 1: < 0.2 g Level 2: 0.2 ~ 0.35 g Level 3: 0.35 ~ 0.5 g Level 4: 0.5 ~ 0.6 g Level 5: > 0.6 g	^56^
5	NDVI	Level 1: 0.0425 ~ 0.5456; Level 2: − 0.0847 ~ 0.0425; Level 3: − 0.2181 ~ − 0.0847; Level 4:-0.4666 ~ − 0.2181; Level 5: − 1 ~ − 0.4666	Natural fracture method
6	Ht	Level 1: > 0.9720; Level 2: 0.9718 ~ 0.9720; Level 3: 0.9716 ~ 0.9718; Level 4: 0.9712 ~ 0.9716; Level 5: < 0.9712	Natural fracture method
7	Lithology	Level 1: hardest (Rb > 60 MPa); Level 2: harder (Rb = 40 ~ 60 MPa); Level 3: softer (Rb = 20 ~ 40 MPa); Level 4: weak (Rb < 20 MP a); Level 5: loose (Unstable rock formation)	

**Figure 6:**
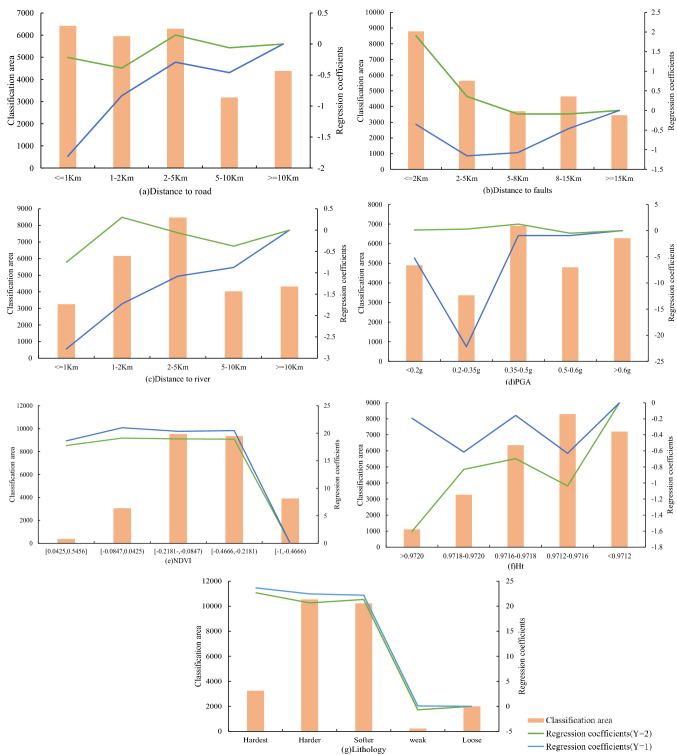
MLR coefficients under the grading of various conditioning factors (Name of software: Office 2019).

The results of the landslide susceptibility prediction model based on KDE-MLR were calculated by SPSS Modeler software. The same sample ratio used in the two-category LR landslide prediction model was used to divide the dataset. In this study, 80% of the dataset was used as the training set (0: 708 individuals in this set, 1: 269 individuals in this set, and 2: 439 individuals in this set) and 20% was used as the validation set (0: 177 individuals in this set, 1: 67 individuals in this set, and 2:110 individuals in this set) (0 represents no landslide point). The dataset comprised 3 categories, with Y = 0 as the reference group. Two groups of regression coefficients with different spatial characteristics of landslides levels were obtained. The results are shown in Fig. 6:

1. When the dependent variable is the moderate spatial characteristics of landslides (Y = 1), lithology and NDVI have the highest effect on the region, and the two variables were positively correlated. The result indicated that the probability of Y = 1 was higher in areas with softer lithology and lower NDVI values compared with the reference group. This implies that areas with less vegetation cover and softer strata were prone to moderate spatial aggregation of landslides. The regression coefficients of each level of the distance to the road, the distance to the fault zone, the distance to the river, PGA, and Ht were all negative values. This finding implies that the intermediate level of spatial characteristics of landslides was negatively correlated with these factors. These results indicate that shorter distance to the road, shorter the distance to the fault zone, and shorter distance to the river are associated with moderated spatial characteristics of landslides are more moderate. In addition, the regression coefficients of PGA and Ht fluctuate according to the classification.
2. When the dependent variable represents the high spatial characteristics of landslides (Y = 2), the regression coefficients of NDVI and lithology are basically consistent with the case of Y = 1. In this case, the high spatial characteristics of landslides exhibited a high dependence on lithology and NDVI, and the spatial characteristics of landslides were positively correlated with lithology and NDVI. The regression coefficient value gradually decreased with increase in the distance to faults. However, all coefficients had positive values, indicating that landslide susceptibility was higher in areas closer to the faults and the influence of this factor on Y = 2 was higher than its influence on Y = 0. The regression coefficients of Ht had negative values, implying a larger value of Ht was associated with a higher correlation of spatial characteristics of landslides. The regression coefficient value of PGA was small, but the change was relatively stable. The regression coefficients of the distance to the road and the distance to the river exhibited distinct correlations according to the classification. The absolute value of the regression coefficient was largest when the distance to the road was 1–2 km and the distance to the river was less than 1 km. The impact on the landslide disaster was largest under these conditions.

The importance of each conditioning factor is obtained by combining the two sets of regression coefficients. From Table 4, it can be concluded that the influence on landslide geological hazards from high to low is PGA, NDVI, lithology, distance to roads, distance to rivers, distance to faults, and Ht.

**Table 4:** The importance of conditioning factors.

Serial number	1	2	3	4	5	6	7
Conditioning factor	Distance to roads	PGA	Distance to rivers	Distance to faults	NDVI	Ht	Litholigy
Predictor importance	0.10	0.42	0.10	0.09	0.12	0.05	0.12

### Performance evaluation of landslide prediction model

The Error matrix in Table 1 is used to calculate the overall accuracy of the KDE-MLR landslide susceptibility prediction model. The results in Table 5 show that the overall accuracy of the training datasets used to build the model is 71%, the overall accuracy of the validation datasets used to verify the model is 70.7%, and the overall accuracy is more than 70%. This demonstrates that the landslide susceptibility prediction model based on KDE-MLR credibly predicts the probability distribution of the spatial aggregation of landslides in the study area^57^. Similarly, the two-category LR landslide prediction model was verified. At the same time, the two-category LR landslide prediction model is verified, and its overall accuracy rate is about 72%, indicating that the two-category LR landslide disaster prediction model is also feasible, because the two models have similar accuracy rates. Therefore, the problem of error caused by model accuracy was excluded in the follow-up study.

**Table 5:** Accuracy results of the susceptibility prediction model of landslides based on KDE-MLR.

	Observed	Predicted			Overall accuracy (%)
		0	1	2	
Training step	0	566	42	100	71.0
	1	116	106	47	
	2	80	26	333	
Validation step	0	132	14	31	70.7
	1	31	26	10	
	2	12	5	93	

### Comparative analysis of landslide susceptibility

**Figure 7:**
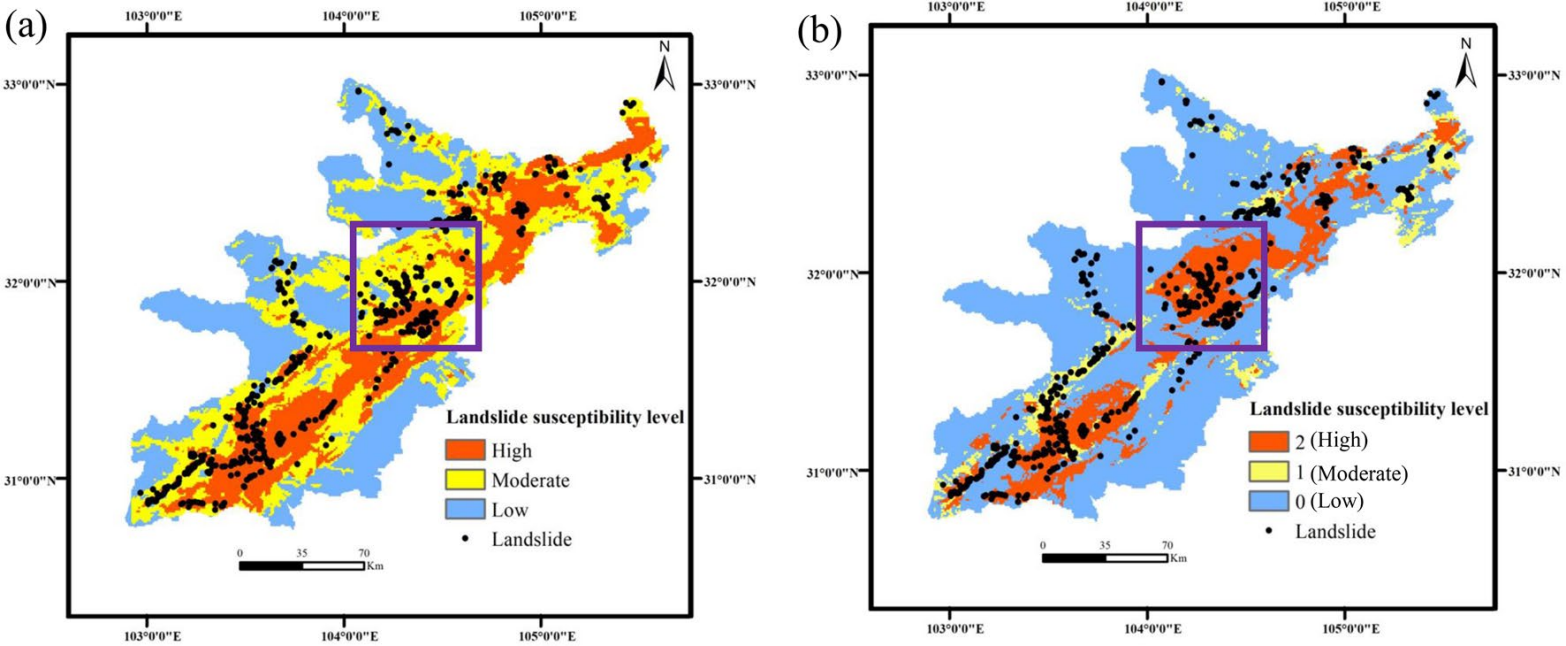
Spatial distribution of the prediction results of landslides in extremely earthquake-stricken areas: (**a**) Landslide prediction distribution map based on the two-category LR-based landslide prediction model; (**b**) Landslide prediction distribution map based on the KDE-MLR model (Names of software: ArcGIS 10.5 and Office 2019).

This paper assigns a corresponding weight value to each conditioning factor based on the regression coefficient. Then, by superimposing each conditioning factor layer, distribution maps of landslide predictions for both the two-category LR-based landslide prediction model (Fig. 7a) and the KDE-MLR model (Fig. 7b) are obtained.

In Table 6 and Fig. 7:

**Table 6:** Forecast result statistics chart.

Model	Forecast result	Number of grids	Area (Km^2^)	Area percentage (%)	Percentage of landslides (%)	Landslide density (10^–3^)
Landslide prediction model based on two-category LR	Low	3,659,903	9640	36.5	9	0.022
	Moderate	3,669,930	9666	36.6	33.2	0.08
	High	2,697,298	7104	26.9	57.8	0.19
The susceptibility prediction model of landslides based on KDE-MLR	0	7,079,154	18,645	70.6	28.8	0.036
	1	882,388	2324	8.8	17.6	0.17
	2	2,065,589	5441	20.6	53.6	0.229

**Figure 8:**
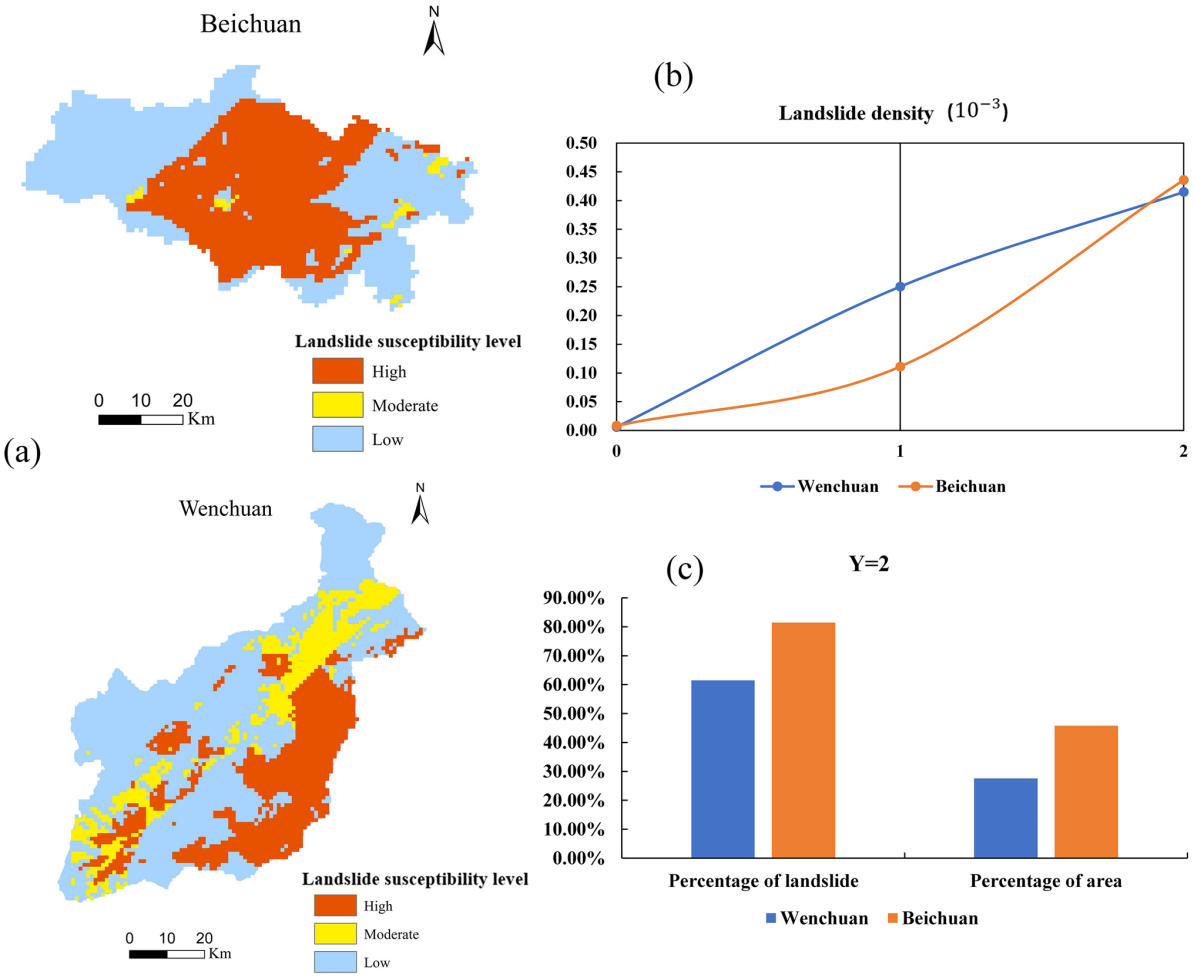
Map of Wenchuan and Beichuan with regional statistical results (Names of software: ArcGIS 10.5 and Office 2019).

1. In the predicted results of the two models above, more than 50% of landslides occur in areas with high landslide susceptibility. In the KDE-MLR model, the area with a landslide susceptibility level of 3 is approximately 5441 km^2^. In the three-level landslide susceptibility area, divided by the two-category LR model according to the natural segment point method, the area of the high-level susceptibility is approximately 7104 km^2^, and the landslide density is significantly lower than the predicted result in the KDE-MLR model. Overall, the landslide susceptibility prediction model based on KDE-MLR constructed in this paper can effectively highlight the spatial characteristics of landslides in 10 extremely earthquake-stricken study areas.
2. The landslide susceptibility prediction model based on KDE-MLR is used to predict the hazard level areas with high spatial characteristics of landslides. According to Fig. 7, it can be seen that most of them are distributed in Beichuan and Wenchuan areas. On the whole, with the increase of susceptibility level, the regional landslide density has also gradually increased. Generally, as the susceptibility level increases, the density of regional landslides also increases. From the statistical analysis shown in Fig. 8, there are a total of 552 landslides in Wenchuan and Beichuan, and approximately 71% of those landslides are in areas with a predicted landslide susceptibility levels of 2. In Beichuan, approximately 80% of the landslides are located in the Y = 2 area, which is more consistent with the spatial distribution of the actual landslide points.

## Discussion

This paper takes 10 extremely earthquake-stricken areas of Wenchuan as examples, constructs a spatial quantitative modelling of landslide of earthquake-stricken area which based on the KDE-MLR. This provides a decision-making basis for the early warning of landslide in earthquake-stricken areas. But it can also be improved from the following aspects:

1. The nonlandslide points in this paper are selected at a certain distance away from the disaster points. According to Zuo and Wang^58^, that the positive training set usually uses known data. However, not all the negative points can be treated as true negative samples, which means that selecting of random negative training points creates uncertainty. For the spatial prediction model of landslides that is driven by supervisory data, identifying negative samples will be the focus of research in the future.
2. When predicting landslides, this paper uses the KDE algorithm to describe the spatial characteristics of landslides. Kristan et al.^59^ proposed a new online estimation method of the probability density function based on kernel density estimation (KDE). This method maintains and updates the nonparametric model of the observed data, and has great advantages over the selection of bandwidth and kernel function in this paper. Additionally, the advantages of machine learning methods in the spatial prediction of landslides are constantly being shown. Therefore, in future research, this paper will consider to using machine learning methods for prediction.

## Conclusions

Taking as examples the landslides in the 10 areas extremely earthquake-stricken areas of the Wenchuan earthquake in 2010, this paper comprehensively considers the spatial location of landslides and other factors after the earthquake and uses the KDE method to quantitatively characterize the spatial characteristics of landslides and improve the intensity description of traditional landslides (the "0–1" occurrence assignment method). Next, the spatial distribution characteristics of landslides after the earthquake are calculated. Then, taking the spatial characteristic of landslides as the dependent variable and each conditioning factor as the independent variable, an MLR model is used to establish a quantitative spatial landslide model. After comparing and analyzing the traditional two-category LR landslides prediction model, the specific conclusions are as follows:

1. In the landslide susceptibility prediction model based on KDE-MLR, the absolute value of the logistic regression coefficient of PGA is the largest (PGA = 0.418), indicating that this conditioning factor has the greatest impact on the landslides in the study area. The value of PGA = 0.42 is basically consistent with these results. NDVI is also has a high value in the KDE-MLR model, however, in the two-category LR model, this conditioning factor has the smallest impact on the prediction results. The predictive variable terrain information entropy is the least important in the KDE-MLR model. The four conditioning factors of lithology, distance to roads, distance to rivers and distance to faults are basically consistent between the two prediction models.
2. The accuracy of the KDE-MLR model when predicting the spatial distribution of landslides in the study area is approximately 70.7%, which is similar to the prediction accuracy of the traditional two-category LR landslide model. However, the landslide prediction susceptibility maps show that the KDE-MLR model susceptibility predictions are more consistent with the spatial distribution of actual landslides. Compared to the landslide prediction results obtained from the two-category LR model, the KDE-MLR model highlights the spatial characteristics of landslides in the 10 extremely earthquake-stricken areas and more accurately expresses the spatial distribution characteristics of landslides. This shows that on the basis of combining GIS technology, the KDE-based, MLR method has great advantages for studying the spatial prediction of earthquake-induced landslides over the traditional, two-category LR method. Furthermore, it has great prospects for promotion and application.

## Data Availability

The datasets used and analysed during the current study available from the corresponding author on reasonable request.

## References

[1] Li X, Li Z, Yang J, Liu Y, Fu B, Qi W, Fan X (2018). Spatiotemporal characteristics of earthquake disaster losses in China from 1993 to 2016. Nat. Hazards.

[2] Han P, Tian S, Fan X, Sheng X (2018). Statistical analysis and forecasting of the secondary disasters induced by lushan earthquake. J. Nat. Disasters.

[3] Ramirez MR, Peek-Asa CL (2005). Epidemiology of traumatic injuries from earthquakes. Epidemiol. Rev..

[4] Dagdelenler G, Nefeslioglu HA, Gokceoglu C (2016). Modification of seed cell sampling strategy for landslide susceptibility mapping: An application from the Eastern part of the Gallipoli Peninsula (Canakkale, Turkey). Bull. Eng. Geol. Environ..

[5] Tsangaratos P, Ilia I (2016). Landslide susceptibility mapping using a modified decision tree classifier in the Xanthi Perfection, Greece. Landslides.

[6] Chen W, Ding X, Zhao R, Shi S (2016). Application of frequency ratio and weights of evidence models in landslide susceptibility mapping for the Shangzhou District of Shangluo City, China. Environ. Earth Sci..

[7] Stahl T, Clark MK, Zekkos D, Athanasopoulos-Zekkos A, Willis M, Medwedeff W, Knoper L, Jin J (2017). Earthquake science in resilient societies. Tectonics.

[8] Xu C, Xu X (2012). Comment on “Spatial distribution analysis of landslides triggered by 2008.5.12 Wenchuan Earthquake, China” by Shengwen Qi, Qiang Xu, Hengxing Lan, Bing Zhang, Jianyou Liu [Engineering Geology 116 (2010) 95–108]. Eng. Geol..

[9] Junsong, L. & Menglan, W. Sensor-based mountain landslide sensitivity and logistics supply chain management optimization. *Arab. J. Geosci.* **14**, 1612.

[10] Pathak D (2016). Knowledge based landslide susceptibility mapping in the Himalayas. Geoenviron. Disasters.

[11] Eslaminezhad SA, Omarzadeh D, Eftekhari M, Akbari M (2021). Development of a data-driven model to predict landslide sensitive areas. Geogr. Tech..

[12] Jennifer JJ, Saravanan S (2021). Artificial neural network and sensitivity analysis in the landslide susceptibility mapping of Idukki district, India. Geocarto Int..

[13] Dikshit A, Sarkar R, Pradhan B, Acharya S, Alamri AM (2020). Spatial landslide risk assessment at phuentsholing, Bhutan. Geosciences.

[14] Schuster RL, Fleming RW (1986). Economic losses and fatalities due to landslides. Environ. Eng. Geosci..

[15] Pollock W, Grant A, Wartman J, Abou-Jaoude G (2019). Multimodal method for landslide risk analysis. MethodsX.

[16] Guzzetti F, Reichenbach P, Ardizzone F, Cardinali M, Galli M (2006). Estimating the quality of landslide susceptibility models. Geomorphology.

[17] Vakhshoori V, Zare M (2016). Landslide susceptibility mapping by comparing weight of evidence, fuzzy logic, and frequency ratio methods. Geomat. Nat. Hazards Risk.

[18] Kritikos T, Robinson TR, Davies T (2015). Regional coseismic landslide assessment without historical landslide inventories: A new approach. J. Geophys. Res. Earth Surf..

[19] Devkota KC, Regmi AD, Pourghasemi HR, Yoshida K, Pradhan B, Ryu IC, Dhital MR, Althuwaynee OF (2013). Landslide susceptibility mapping using certainty factor, index of entropy and logistic regression models in GIS and their comparison at Mugling-Narayanghat road section in Nepal Himalaya. Nat. Hazards.

[20] Regmi AD, Devkota KC, Yoshida K, Pradhan B, Pourghasemi HR, Kumamoto T, Akgun A (2014). Application of frequency ratio, statistical index, and weights-of-evidence models and their comparison in landslide susceptibility mapping in Central Nepal Himalaya. Arab. J. Geosci..

[21] Youssef AM, Al-Kathery M, Pradhan B (2015). Landslide susceptibility mapping at Al-Hasher area, Jizan (Saudi Arabia) using GIS-based frequency ratio and index of entropy models. Geosci. J..

[22] Chen W, Panahi M, Pourghasemi HR (2017). Performance evaluation of GIS-based new ensemble data mining techniques of adaptive neuro-fuzzy inference system (ANFIS) with genetic algorithm (GA), differential evolution (DE), and particle swarm optimization (PSO) for landslide spatial modelling. CATENA.

[23] Pradhan B, Lee S (2010). Landslide susceptibility assessment and factor effect analysis: Backpropagation artificial neural networks and their comparison with frequency ratio and bivariate logistic regression modelling. Environ. Model. Softw..

[24] Xu C, Dai F, Xu X, Lee YH (2012). GIS-based support vector machine modeling of earthquake-triggered landslide susceptibility in the Jianjiang River watershed, China. Geomorphology.

[25] Teshale AB (2022). Factors associated with unmet need for family planning in sub-Saharan Africa: A multilevel multinomial logistic regression analysis. PLoS ONE.

[26] Bai SB, Wang J, Lü GN, Zhou PG, Hou SS, Xu SN (2010). GIS-based logistic regression for landslide susceptibility mapping of the Zhongxian segment in the Three Gorges area, China. Geomorphology.

[27] Bui DT, Lofman O, Revhaug I, Dick O (2011). Landslide susceptibility analysis in the Hoa Binh province of Vietnam using statistical index and logistic regression. Nat. Hazards.

[28] Tobler W (1983). An alternative formulation for spatial-interaction modeling. Environ. Plan. A.

[29] Zou F, Zhan Q, Zhang W (2018). Quantifying the impact of human activities on geological hazards in mountainous areas: Evidence from shennongjia. China. Nat. Hazards..

[30] Liu B, Chen X, Zhou Z, Tang M, Li S (2020). Research on disaster resilience of earthquake-stricken areas in Longmenshan fault zone based on GIS. Environ. Hazards.

[31] Liu Y, Yuan X, Liang G, Huang Y, Zhang X (2017). Driving force analysis of the temporal and spatial distribution of flash floods in Sichuan province. Sustainability.

[32] Gonzalez HR, Mora CJC, Aguirre GJ, Novelo CDA (2013). The velocity structure and its relationship to seismic hazard in Tuxtla Gutierrez, Chiapas. Rev. Mex. Cienc. Geol..

[33] Wu, C. S. Study on the optimization of ecological restoration monitoring in the severely affected areas of Wenchuan Earthquake. *Hunan Univ. Sci. Technol*. (2013) (**in Chinese**).

[34] Shen P, Zhang LM, Fan RL, Zhu H, Zhang S (2020). Declining geohazard activity with vegetation recovery during first ten years after the 2008 Wenchuan earthquake. Geomorphology.

[35] Crozier MJ (1986). Landslides: Causes, Consequences and Environment.

[36] Ayalew L, Yamagishi H, Marui H, Kanno T (2005). Landslides in Sado Island of Japan: Part II. GIS-based susceptibility mapping with comparisons of results from two methods and verifications. Eng. Geol..

[37] Anderson M, Glade T, Crozier M (2005). landslide and Risk.

[38] Li, Y. Q. Geological Architecture and Formation Mechanism of the transitional zone between Longmenshan Mountains and Sichuan Basin. Doctoral dissertation, China University of Geosciences (Beijing). https://kns.cnki.net/KCMS/detail/detail.aspx?filename=1018029944.nh&dbname=CDFDTEMP (2018) (**in Chinese**).

[39] Xu Q, Liu CL, Zhang B, Liang N, Tong LQ (2009). Slope instabilities in the severest disaster areas of 5.12 Wenchuan earthquake. J. Eng. Geol..

[40] Parzen E (1962). On estimation of a probability density function and mode. Ann. Math. Stat..

[41] Chong XU, Dai FC, Su-Ning XU, Xi-Wei XU, Hong-Lin HE, Xi-Yan WU, Shi F (2013). Application of logistic regression model on the Wenchuan earthquake triggered landslide hazard mapping and its validation. Hydrogeol. Eng. Geol..

[42] Dai FC, Lee CF, Li J, Xu ZW (2001). Assessment of landslide susceptibility on the natural terrain of Lantau Island, Hong Kong. Environ. Earth Sci..

[43] Ayalew L, Yamagishi H (2005). The application of GIS-based logistic regression for landslide susceptibility mapping in the Kakuda-Yahiko Mountains, Central Japan. Geomorphology.

[44] Mathew J, Jha VK, Rawat GS (2009). Landslide susceptibility zonation mapping and its validation in part of Garhwal Lesser Himalaya, India, using binary logistic regression analysis and receiver operating characteristic curve method. Landslides.

[45] Conoscenti C, Angileri S, Cappadonia C, Rotigliano E, Agnesi V, Märker M (2014). Gully erosion susceptibility assessment by means of GIS-based logistic regression: A case of Sicily (Italy). Geomorphology.

[46] Achu AL, Aju CD, Reghunath R (2020). Spatial modelling of shallow landslide susceptibility: A study from the southern Western Ghats region of Kerala, India. Ann. GIS Geogr. Inf. Sci..

[47] Luo YD, Lu L (2021). Network attack detection based on artificial neural network and genetic algorithm. Comput. Eng. Des..

[48] Gortmaker SL, Hosmer DW, Lemeshow S (1994). Applied logistic regression. Contemp. Sociol..

[49] Long JS (1997). Regression Models for Categorical and Limited Dependent Variables.

[50] Chan HC, Chang CC, Chen PA, Lee JT (2019). Using multinomial logistic regression for prediction of soil depth in an area of complex topography in Taiwan. CATENA.

[51] Story M, Congalton RG (1986). Accuracy assessment: A user’s perspective. Photogramm. Eng. Remote Sens..

[52] Wright RE, Grimm LG, Yarnold PR (1995). Logistic regression. Reading and Understanding Multivariate Statistics.

[53] Yan YQ, Yang ZH, Zhang XJ, Meng SW, Guo CB, Wu R, Zhang YY (2021). Landslide susceptibility evaluation of Batang fault zone in eastern Qinghai-Tibet Plateau based on weighted weight of evidence model. Mod. Geol..

[54] Liu J, Li S, Chen T (2018). Landslide susceptibility evaluation based on optimized random forest model. J. Wuhan Univ. (Information Science Edition).

[55] Liu FZ, Wang L, Xiao D (2021). Application of machine learning model in landslide susceptibility evaluation. Chin. J. Geol. Hazard Prev..

[56] Liu R, Li LY, Yang X, Yang YT, Yang M (2021). Landslide susceptibility evaluation based on factor strong correlation analysis method. Earth Environ..

[57] Landis JR, Koch GG (1977). The measurement of observer agreement for categorical data. Biometrics.

[58] Zuo R, Wang Z (2020). Effects of random negative training samples on mineral prospectivity mapping. Nat. Resour. Res..

[59] Kristan M, Leonardis A, Skocaj D (2011). Multivariate online kernel density estimation with Gaussian kernels. Pattern Recogn..

